# Implication of problematic substance use in poststroke depression: an hospital-based study

**DOI:** 10.1038/s41598-021-92639-5

**Published:** 2021-06-25

**Authors:** Yolaine Rabat, Igor Sibon, Sylvie Berthoz

**Affiliations:** 1 Univ. Bordeaux, CNRS, EPHE, INCIA, UMR 5287, 33000 Bordeaux, France; 2 Stroke Unit, Department of Neurology, CHU Bordeaux, Bordeaux, France; 3 Department of Psychiatry for Adolescents and Young Adults, Institut Mutualiste Montsouris, Paris, France

**Keywords:** Stroke, Risk factors, Epidemiology, Population screening

## Abstract

The prevalence of clinically defined problematic substance use among stroke patients is overlooked and its association with post-stroke depression (PSD) is unknown. Our aims were to: (1) estimate the proportion of stroke patients with a problematic substance use as defined by clinical screening scales; (2) determine the proportion of PSD at three months of follow-up; (3) explore if the baseline severity in substance use and its evolution are independent predictors of PSD. A cohort of first-ever non-severe stroke adult patients was screened at baseline and three months post-stroke using recommended cut-off scores of standardized scales for tobacco, alcohol and cannabis abuse. PSD was defined using the Center of Epidemiological Studies Depression scale score. Out of the 244 eligible patients, 74 (30.3%) presented a problematic substance use, including 21 (8.6%) polydrug abusers. Among these patients, the prevalence of PSD was 50.8%, including 29.5% of severe depression. The severity of tobacco dependence at baseline was found to double the risk (*OR* 1.59, 95% *CI* 1.05–2.43) of presenting a PSD, independently of previously reported risk factors. We found no significant evidence for an effect of the evolution in substance use at follow-up. Addictive disorders are part of the critical unmet needs that should be addressed in the management of PSD.

## Introduction

Approximatively one-third of stroke patients will experience mood impairments, notably depression, during follow-up^1^*.* This frequent complication is an independent predictor of poor functional outcome, impaired quality of life, stroke recurrence and mortality^2–5^*.* According to recent meta-analyses, the most consistent predictors of post-stroke depression (PSD) are higher functional disability, stroke severity, cognitive impairment, personal history of a mood disorder and a lack of social support, while hemisphere or lesion location or subtype of stroke, age and gender have been less consistently reported to be associated with an higher risk of PSD^6–8^. These meta-analyses also suggest there are different risk factors for PSD at different time points after the stroke.

Independently of stroke, poor lifestyle habits such as chronic tobacco, alcohol and cannabis consumption and related dependence—which can be referred to as problematic substance use (PSU) or addiction disorders -, are known to be tightly associated with depression, in particular during substance use cessation treatment^9^. Smoking, drinking and illicit drug consumption are among the most common modifiable life-style stroke risk factors. Although these lifestyle habits are usually reported in stroke studies^10^, related clinically defined problematic substance use are unfrequently evaluated in clinical practice while their presence might represent a potential marker of psychological fragility and preclude the ability to achieve lifestyle changes that are requested following stroke.

Moreover, the relationships between problematic substance use and PSD remain overlooked. We searched PubMed from MEDLINE and Scopus and Google scholar for original articles for the following indication terms: (addiction OR alcohol OR tobacco OR cannabis OR substance use disorder OR life-style OR secondary prevention OR behavior change) AND (stroke OR post-stroke outcome OR post-stroke) AND (depression OR mood disorder OR mood impairment). Searches were restricted to articles published in English. One author (Y.R) conducted the data extraction and collected the articles, and two authors (Y.R and S.B) independently inspected the abstracts and discarded those that were not discernibly on the predictors of depression after stroke. The remaining articles were extracted and scrutinized. From an initial set of over 1000 references that came out, we failed to identify one that examined the impact of clinically defined substance use disorder/addiction on PSD.

To the best of our knowledge, for now, the strongest argument driving the move towards considering problematic substance use as a potential risk factor for PSD appeared in Perrain et al.’s meta-analysis on the clinical risk factors of PSD^11^. Tobacco consumption (*yes*, *no*) was identified as a significant predictor with a mean *RR* 1.36 (*95% CI* 0.86–2.16), and was ranked just after cognitive impairment *RR*:1.52; (*95% CI* 0.59–3.96). In this review, alcohol consumption had a mean RR < 1: 0.83 (*95% CI* 0.49–1.42) and with some potential biases.

To contribute to this field of research and fill gaps in the literature, we conducted a monocentric hospital-based study. Our aims were: (1) to describe in a stroke population the proportion of patients with a problematic substance use as defined by recommended cut-off scores of clinical screening scales for addictive behaviors; (2) to determine the proportion of patients with a problematic substance use presenting a PSD at three months of follow-up; (3) to explore if the baseline severity of the problematic substance use and the evolution in substance use following stroke predicts PSD independently of well-known PSD risk factors.

## Methods

### Study design

This was a retrospective analysis of a prospectively compiled database of consecutive patients admitted to the comprehensive stroke unit of the Bordeaux University Hospital from January to June 2019. This stroke unit admits patients living in close proximity and some patient referred by primary hospitals of the region for endovascular therapy.

We included patients between 18 and 85 years of age with a first ever acute stroke (symptoms onset from less than 15 days) confirmed on brain imaging (MRI or CT) with available data from electronic medical record at admission and three months follow-up. Exclusion criteria were severe stroke as indexed by a score ≥ 18 at the National Institutes of Health Stroke Scale (NIHSS;^12^); severe aphasia or cognitive impairment (Montreal Cognitive Assessment—MoCA ≤ 14;^13^) impeding answers to the questionnaires, or inability to fluently read/speak French.

### Standard protocol approval and patients consent

The study population is part of the ObA2 regional cohort (National commission for data protection CNIL authorization n°911201). Each patient provided oral informed consent for the use of clinical, biological and imaging data as collected in standard care. All methods were carried out in accordance with relevant guidelines and regulations. In addition, the present study has been validated by the local ethical board of the Bordeaux University Hospital (GP-CE-2020-13).

### Assessment

Demographic and clinical data including, vascular risk factors, stroke subtypes, stroke severity (NIHSS) and stroke outcome (modified Rankin scale—mRS, which categorizes the level of functional independence) were collected. Patients were asked about tobacco, alcohol and cannabis consumption as a standard evaluation of the vascular risk factors.

When symptoms about problematic substance use of alcohol, cannabis or tobacco were suspected during the routine care assessment, a more detailed evaluation of dependence to these substances was performed using standardized clinical scales that have been validated and as recommended by the French health Authority (HAS):

- Nicotine dependence was assessed using the Fagerström Test for Nicotine Dependence (FTND)^14^. Scores can range from zero to ten. The recommended cut-off scores to index the categories of nicotine dependence severity are as follows: below two = no dependence, three to four = mild severity, five to six = moderate severity, seven to ten = extreme severity. Accordingly, patients with a score greater than two were considered to have a problematic tobacco use (PTU).
- Alcohol dependence was measured using the Alcohol Use Disorder Identification Test (AUDIT)^15^. Scores can range from zero to 40. The recommended cut-off scores to index the categories of alcohol dependence severity are as follows: from six to 12 for women and seven to 12 for men = moderate severity, above 12 = extreme severity. Accordingly, women with a score greater than five and men with a score greater than six were considered to have a problematic alcohol use (PAU).
- Cannabis dependence was assessed using the Cannabis Abuse Screening Test (CAST)^16^. Scores can range from zero to six. The recommended cut-off scores to index the categories of cannabis dependence severity are as follows: below two = low risk of dependence, a score of two = moderate risk of dependence, three or more = high risk of dependence. Accordingly, patients with a score of two or more were considered to have a problematic cannabis use (PCU).
- Polysubstance use disorder was defined when a patient scored positively for at least two substances.

In addition, current and/or past major depressive episode was evaluated using the Mini-International Neuropsychiatric Interview (MINI 5.0.0)^17^.

At the three months follow-up visit, in addition to the conventional evaluations (NIHSS, mRS, MoCA), patients completed the same problematic substance use screening scales and the Center of Epidemiological Studies Depression scale (CESD)^18^. CESD scores can range from zero to 60 and index the following categories of depression severity: below 16 for no depression; 16 to 22 for moderate depression; above 23 for severe depression.

Finally, evolution in substance use at follow-up was dichotomized into a binary category depending on whether the patients managed versus failed to follow the recommendation, i.e. stopped their consumption or decreased their problematic substance use or not (Better Life-style Habits vs No Better Life-style Habits).

### Statistical analyses

The primary research question of the paper and analysis plan are not been pre-registered on a publicly available platform, prior to undertaking the analysis.

Quantitative variables were described using means, standard deviations (SD) and range values. Categorial variables were described using counts and proportions.

Psychological status at follow-up was dichotomized into a binary category (PSD^+^ vs PSD^-^) where PSD^+^ sub-group included patients with moderate and severe depression. Between-group comparisons depending on the presence or not of a PSD at follow-up were conducted using Mann–Whitney and Chi2 or Fischer tests for, respectively, quantitative and categorial variables.

The association between the baseline severity of the problematic substance use and PSD at three months, independently of known PSD risk factors, was tested using multivariate logistic regression models. This was conducted among the problematic tobacco use sub-group only, the problematic alcohol use and problematic cannabis use samples’ sizes being not large enough to provide an adequate power to detect statistical significance. Two models were built to test the association between the baseline severity of the problematic tobacco use and PSD. Model 1 included the baseline variables that reached a *p* < 0.05 in the univariate analyses and variables reported in the literature to predict PSD such as age, gender, psychiatric history, and activities of daily living (mRS). The same methodology was used for Model 2, but using the three months follow-up variables reported to be associated with PSD (mRS and MoCA) as well as the evolution in substance use (Better Life-style Habits).

*p* < 0.05 was considered statistically significant. All the statistical analyses were conducted using Jamovi 1.1.9.

### Statement of ethics

Patients provided oral informed consent. All methods were carried out in accordance with relevant guidelines and regulations. No personal details are included in any parts of the manuscript. All data was obtained in routine care in agreement with the Bordeaux University Hospital ethical review committee (approval ref GP-CE-2020-13). All the participants of the study were included in the “Observatoire Aquitain des AVC3” (ObA2 registry) supervised by the regional health agency of Nouvelle Aquitaine and having the requested authorization (National commission for data protection CNIL n°911201). The study protocol and procedure complied with the Helsinki declaration.

## Results

**Figure 1:**
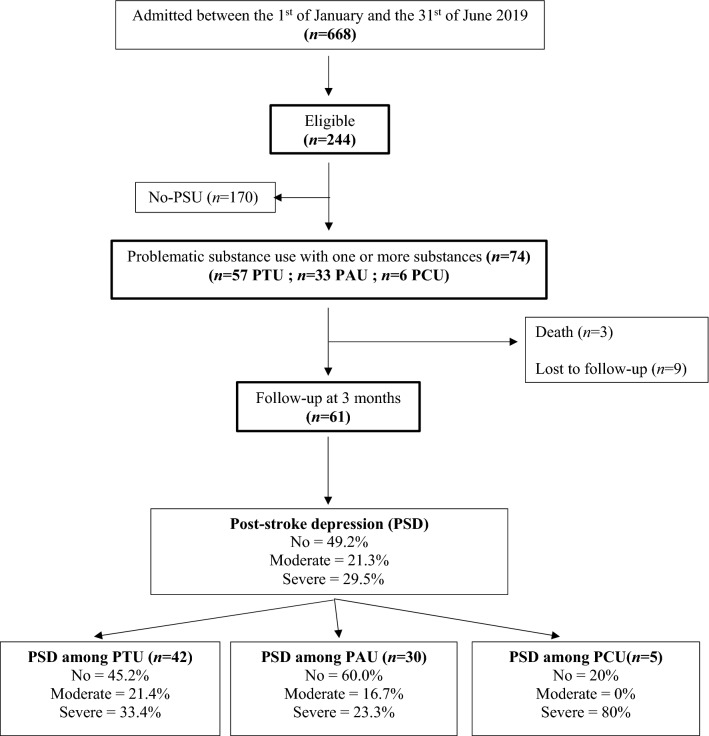
Study flow-chart. *PAU* Problematic Alcohol Users, *PCU* Problematic cannabis users, *PSU* Problematic Substance Users, *PTU* Problematic Tobacco Users.

The study flow-chart is illustrated in Fig. 1.

### Problematic substance use prevalence

Among the 244 eligible patients, 74 (30.3%) had a problematic substance use at baseline, problematic tobacco use being the most frequent (77%). Severe problematic substance use for tobacco, alcohol or cannabis concerned respectively 34.0%, 18.2% and 60.0% of the samples. There were 21 polysubstance abusers: 15 were tobacco and alcohol abusers, 5 were tobacco and cannabis abusers, and only one had a problematic substance use for the 3 substances.

Socio-demographic and clinical characteristics of the patients with a problematic substance use are presented in Tables 1 and 2. All but 9 (91.8%) had an ischemic stroke. Regarding psychiatric history, 14.9% and 10.8% endorsed a diagnosis of, respectively, a past and a current depression at baseline.

**Table 1:** Characteristics of the overall sample at baseline.

Demographic & Clinical characteristics	Baseline values (*n* = 74)	CESD defined post-stroke depression at three months (N = 61)		Statistics; *p* (*n*)
		PSD− (*n* = 30)	PSD+ (*n* = 31)	
Male, *n* (%)	51 (68.9%)	25 (41.0%)	20 (32.8%)	0.095 (61)
Age, *m* ± *SD* (range)	60.3 ± 12 (23–84)	59.6 ± 13.1 (23–78)	61.3 ± 12.6 (36–84)	0.862(61)
NIHSS at admission, *m* ± *SD* (range)	2.73 ± 3.13 (0–17)	2.20 ± 2.71 (0–13)	3.45 ± 3.56 (0–17)	0.057(61)
MoCA at admission, *m* ± *SD* (range)	22.7 ± 5.19 (3–29)^a^	22.6 ± 5.47 (3–29)	23.9 ± 4.09 (14–29)	0.444 (52)
MoCA at three months, *m* ± *SD* (range)	26.5 ± 3.14 (11–29)^b^	26.0 ± 3.75 (11–29)	27.1 ± 2.39 (19–29)	0.213 (57)
mRS at hospitalization, *m* ± *SD* (range)	1.19 ± 1.20 (0–5)	1.17 ± 1.09 (0–4)	1.16 ± 1.32 (0–5)	0.711 (61)
mRS at three months, *m* ± *SD* (range)	1.31 ± 0.90 (0–3)^c^	1.00 ± 0.79 (0–3)	1.60 ± 0.93 (0–3)	0.13 (60)
Past or current major depression,* n* (%)	16 (21.6%)	3 (4.92%)	9 (9.84%)	0.106 (61)
Past depression at admission,* n* (%)	11 (14.9%)	2 (3.3%)	5 (8.2%)	
Current depression at admission,* n* (%)	8 (10.8%)	1 (1.6%)	6 (9.8%)	
FTND at admission, *m* ± *SD* (range)	5.91 ± 1.94 (3–10) ^d^	5.21 ± 1.93 (3–10)	6.35 ± 1.82 (3–10)	0.035 (42)
AUDIT at admission, *m* ± *SD* (range)	10.6 ± 5.67 (6–33) ^e^	9.94 ± 4.71 (6–26)	11.0 ± 7.15 (37–33)	0.684 (30)
CAST at admission, *m* ± *SD* (range)	3.80 ± 1.48 (2–6) ^f^	2.00 ± Na	3.75 ± 1.71 (2–6)	NA (5)
**Vascular risk factors (admission), *n* (%)**				
Hypertension	34 (45.9%)	15 (24.6%)	15 (24.6%)	0.900 (61)
Dyslipidemia	26 (35.1%)	7 (11.5%)	14 (23.0%)	0.073 (61)
Diabetes	14 (18.9%)	5 (8.20%)	7 (11.5%)	0.561 (61)
Obesity	14 (18.9%)	7 (11.7%)	4 (6.7%)	0.335 (61)
**Problematic substance use**				
Tobacco *(n*, %)	57 (77%)	19 (45.2%)	23 (54.8%)	0.510 (42)
Mild severity	14 (25.9%)	7 (16.7%)	4 (9.5%)	
Moderate severity	22 (40.7%)	8 (19.0%)	10 (23.8%)	
Extreme severity	18 (33.3%)	4 (9.6%)	9 (21.4%)	
Alcohol (*n*, %)	33 (44.6%)	18 (60.0%)	12 (40.0%)	0.096 (30)
Moderate severity	27 (81.8%)	15 (50.0%)	10 (33.3%)	
Extreme severity	6 (18.2%)	3 (10.0%)	2 (6.7%)	
Cannabis (*n*, %)	6 (8.2%)	1 (20.0%)	4 (80.0%)	NA (5)
Drug use profile (*n*, %)	74 (100%)	30 (70.1%)	31 (50.9%)	0.934 (61)
Mono-substance abusers	53 (71.6%)	21 (34.4%)	22 (36.2%)	
Poly-substance abusers	21 (28.4%)	9 (14.7%)	9 (14.7%)	

*AUDIT* Alcohol Use Disorders Identification Test, *CAST* Cannabis Abuse Screening Test, *FTND* Fagerström Test for Nicotine Dependence, *CESD* Center of Epidemiological Studies Depression scale, *MoCA* Montreal Cognitive Impairment, *mRS* modified Rankin Scale, *NIHSS* National Institutes of Health Stroke Scale, *PSD*^+^ Sub-group of patients with CESD scores indexing a moderate or severe depression, *PSD*^*−*^ Sub-group of patients with CESD scores indexing no depression.

^a^(*n* = 62), ^b^(*n* = 58), ^c^(*n* = 61), ^d^(*n* = 54), ^e^(*n* = 33), ^f^(*n* = 5).

**Table 2:** Characteristics of the sub-group of patients with a problematic substance use.

Baseline values (*n* = 74)	Socio-demographic and clinical characteristics	Baseline characteristics (*N* = 57)	CESD defined post-stroke depression at three months (*n* = 42)		Statistics p (*n*)
			PSD− (*n* = 19)	PSD+ (*n* = 23)	
Problematic tobacco use (*n* = 57/74; 77%)	Male, *n* (%)	37 (64.9%)	17 (40.5%)	13 (31.0%)	0.019 (42)
	Age, *m* ± *SD* (range)	58.2 ± 11.4 (23–80)	60.11 ± 11.29 (40–78)	57.35 ± 11.48 (36–80)	0.390 (42)
	NIHSS at admission *m* ± *SD* (range)	2.81 ± 3.31 (0–17)	2.47 ± 3.15 (0–13)	3.65 ± 3.73(0–17)	0.121 (42)
	MoCA at admission *m* ± *SD* (range)	22.4 ± 5.57 (3–29) ^a^	21.63 ± 5.97 (3–29)	23.71 ± 4.06 (14–29)	0.258 (36)
	MoCA at three months *m* ± *SD* (range)	26.2 ± 3.47 (11–29) ^b^	25.32 ± 4.33 (11–29)	26.90 ± 2.63 (19–29)	0.181 (40)
	mRS at hospitalization, *m* ± *SD* (range)	1.18 ± 1.20 (0–4)	1.32 ± 1.25 (0–4)	1.17 ± 1.32 (0–5)	0.382 (42)
	mRS at three months *m* ± *SD* (range)	1.42 ± 0.87 (0–3) ^c^	1.32 ± 0.75 (0–3)	1.57 ± 0.98 (0–3)	0.311 (41)
	Past or current major depression, *n* (%)	15 (26.3%)	2 (4.8%)	9 (21.4%)	0.075 (42)
	Past depression at admission,* n* (%)	11 (14.9%)	1 (2.4%)	5 (11.9%)	–
	Current depression at admission,* n* (%)	8 (10.8%)	1 (2.4%)	6 (14.3%)	
	FTND at admission, *m* ± *SD* (range)	5.91 ± 1.94 (3–10)	5.21 ± 1.93 (3–10)	6.35 ± 1.82 (3–10)	0.035 (42)
	**Known risk factors (admission), *n* (%)**				
	Hypertension	23 (40.4%)	8 (19.0%)	10 (23.8%)	0.929 (42)
	Dyslipidemia	18 (31.6%)	5 (11.9%)	9 (21.4%)	0.381 (42)
	Diabetes	9 (15.8%)	4 (9.5%)	3 (7.1%)	NA (42)
	Obesity	7 (12.3%)	3 (7.1%)	2 (4.8%)	NA (42)
	**Problematic substance use**				
	Tobacco severity (*n*, %)	57 (100)	19 (45.2%)	23 (54.8%)	0.510 (42)
	Mild severity	14 (25.9%) ^d^	7 (16.7%)	4 (9.5%)	–
	Moderate severity	22 (40.7%) ^d^	8 (19.0%)	10 (23.8%)	
	Extreme severity	18 (33.3%) ^d^	4 (9.6%)	9 (21.4%)	
	Drug use profile (*n*, %)	57 (100)	19 (45.2%)	23 (54.8%)	0.879 (42)
	Mono-substance abusers	36 (63.2%)	12 (28.6%)	14 (33.3%)	–
	Poly-substance abusers	21 (36.8%)	7 (16.7%)	9 (21.4%)	

*CESD* Center of Epidemiological Studies Depression scale, *FTND* Fagerström Test for Nicotine Dependence, *AUDIT* Alcohol Use Disorders Identification Test, *CAST* Cannabis Abuse Screening Test, *MoCA* Montreal Cognitive Impairment, *mRS* modified Rankin Scale, *NIHSS* National Institutes of Health Stroke Scale, *PSD*^+^ Sub-group of patients with CESD score indexing a moderate or severe depression, *PSD*^*−*^ Sub-group of patients with CESD score indexing no depression.

a (*n* = 48); b (*n* = 44); c (*n* = 45); d (*n* = 54);

### Prevalence of post-stroke depression among the problematic substance use

Based on the CESD scores collected at follow-up (82.4%; Fig. 1), and independently of the substance, the prevalence of PSD was 50.8%, including 29.5% of severe PSD.

PSD prevalence by substance was 54.8%, 40.0% and 80% for the problematic tobacco use, problematic alcohol use and problematic cannabis use respectively. Half of the poly-substance abusers presented a PSD.

### Evolution in substance use in the overall sample (Table 3)

**Table 3:** Evolution in substance use by substance use disorder group and proportion of PSD in each group.

SUD			Baseline Characteristics		CESD defined post-stroke depression at three months				Statistics p (*n*)
Tobacco evolution (***n***, %)			(***n*** = 57)		PSD^-^ (***n*** = 19)		PSD+ (***n*** = 23)		BLH^+^/BLH^-^ 0.349 (39) (df = 1) – Stop/Dec/ NoCh/Inc NA (39) (df = 3)
Better life style habits at three months		Stopped their consumption	38 (88.4%)^a^	24 (55.8%)^a^	17 (43.6%)	7 (17.9%)	17 (43.6%)	13 (33.3%)	
		Decreased their PSU		14 (32.6%)^a^		10 (25.6%)		4 (10.3%)	
No better life style habits at three months		No change in consumption	5 (11.6%)^a^	4 (9.3%)^a^	1 (2.6%)	1 (2.6%)	4 (10.3%)	3 (7.7%)	
		Increased their PSU		1 (2.3%)^a^		0 (0%)		1 (2.6%)	
Alcohol evolution (***n***, %)			(***n*** = 33)		PSD− (***n*** = 18)		PSD+ (***n*** = 12)		BLH^+^/BLH^-^ 1.000 (30) (df = 1) – Stop/Dec/ NoCh/Inc NA (30) (df = 3)
Better life style habits at three months	Stopped their consumption		22 (73.3%)^b^	6 (20.0%)^b^	13 (43.3%)	2 (6.7%)	9 (30.0%)	4 (13.3%)	
	Decreased their PSU			16 (53.3%) ^b^		11 (36.7%)		5 (16.7%)	
No better life style habits at three months	No change in consumption		8 (26.7%)^b^	8 (26.7%)^b^	5 (16.7%)	5 (16.7%)	3 (10.0%)	3 (10.0%)	
	Increased their PSU			0 (0.0%)^b^		0 (0.0%)		0 (0.0%)	
Cannabis evolution (***n***, %)			(***n*** = 6)		PSD− (***n*** = 1)		PSD+ (***n*** = 4)		NA (5)
Better life style habits at three months	Stopped their consumption		3 (50.0%)	3 (50.0%)	1 (20%)	1 (20%)	1 (20%)	1 (20%)	
	Decreased their PSU			0 (0.0%)		0 (0%)		0 (0%)	
No better life style habits at three months	No change in consumption		3 (50.0%)	2 (33.3%)	0 (0%)	0 (0%)	3 (60%)	2 (40.0%)	
	Increased their PSU			1 (16.7%)		0 (0%)		1 (20.0%)	

*SUD* Substance use disorder, *BLH*^+^ Better life style habits at three months, *BLH*^*−*^ No better life style habits at three months

^a^(*n* = 43), ^b^(*n* = 30).

At three months of follow-up, 55.8% of the problematic tobacco use, 20.0% of the problematic alcohol use and 50.0% of the problematic cannabis use reported having stopped their consumption. Clinically-defined substance use severity decreased for 32.6% of the problematic tobacco use, 53.3% of the problematic alcohol use; it remained unchanged for 9.3% of the problematic tobacco use, 26.5% of the problematic alcohol use and 33.3% of the problematic cannabis use; and increased for 2.3% problematic tobacco use and 16.7% problematic cannabis use.

In sum, Better Life-style Habits concerned: 88.4% of the problematic tobacco use group; 73.3% of the problematic alcohol use group and 50% of the problematic cannabis use group. It concerned 82.5% of the mono-substance abusers and 57.9% of the poly-substance abusers.

### PSD depending on the evolution in substance use (Table 3)

Among the patients who achieved Better Life-style Habits, PSD prevalence was 50.0% of the problematic tobacco use group, 40.9% of the problematic alcohol use group, 50.0% of the problematic cannabis use group.

Among those who did not achieve Better Life-style Habits, PSD prevalence was, 80.0% among the problematic tobacco use group, 37.5% among the problematic alcohol use group, and 100.0% among the problematic cannabis use group.

### PSD^+^ vs PSD^−^ comparison

The level of tobacco dependence at admission and the mRS at three months of follow-up scores differed significantly between the two groups, the PSD^+^ group endorsing higher scores (respectively *p* = 0.032 and *p* = 0.035). No other variable was significantly different (Table 1).

In the sub-group with a problematic tobacco use (problematic tobacco use, Table 2), female gender was more frequent and levels of tobacco dependence at admission were higher among the PSD^+^ than the PSD^-^ (respectively *p* = 0.019; *p* = 0.035).

### Multivariate logistic regressions among the problematic tobacco use (Table 4)

**Table 4:** Predictors of three months post-stroke depression among patients with a problematic tobacco use.

Predictor	Estimate	SE	Z	p	Odds ratio	CI 95%
Model 1a						
Age	0.01	0.04	0.31	0.75	1.01	0.95–1.09
Gender	1.83	1.01	1.81	0.07	6.24	0.86–45.19
History of depression	1.47	0.91	1.62	0.11	4.33	0.73–25.69
mRS_hosp	− 0.07	0.29	− 0.22	0.82	0.94	0.53–1.67
Model 1b						
Age:	0.01	0.04	0.37	0.71	1.01	0.94–1.10
Gender	2.56	1.16	2.21	0.03	12.89	1.33–124.43
History of depression	1.42	0.97	1.46	0.14	4.15	0.62–27.98
mRS_hosp	− 0.04	0.31	− 0.12	0.90	0.96	0.52–1.77
FTND	0.47	0.21	2.16	0.03	1.59	1.05–2.43
Goodness-of-fit indices	Model	Deviance	AIC	AICc	BIC	R^2^N
	Model 1a	48.5	58.5	60.1	67.2	0.27
	Model 1b	42.8	54.8	57.2	65.2	0.40
	Model 1a–1b comparison: X^2^ = 5.71; p = 0.017					
Model 2a						
Age	0.05	0.05	0.99	0.32	1.05	0.95–1.16
Gender	2.09	1.08	1.93	0.05	8.08	0.97–67.42
mRS_three_months	0.69	0.51	1.33	0.18	2.00	0.72–5.53
MoCA_three_months	0.14	0.13	1.13	0.26	1.15	0.90–1.48
History of depression	1.53	1.01	1.52	0.13	4.63	0.64–33.33
Model 2b						
Age	0.06	0.06	1.07	0.28	1.06	0.95–1.18
Gender	3.30	1.41	2.32	0.02	26.98	1.68–433.05
mRS_three_months	0.30	0.60	0.50	0.62	1.35	0.42–4.34
MoCA_three_months	0.32	0.15	2.10	0.04	1.37	1.02–1.84
History of depression	1.54	1.17	1.30	0.19	4.65	0.47–46.45
FTND	0.73	0.30	2.43	0.02	2.08	1.15–3.74
Goodness-of-fit indices	Model	Deviance	AIC	AICc	BIC	R^2^N
	Model 2a	43.2	55.2	57.8	65.3	0.35
	Model 2b	34.7	48.7	52.2	60.5	0.54
	Model 1a–1b comparison: *X*^*2*^ = 8.54; *p* = 0.003					

Model 1. Impact of hospitalization variables on the prediction of post-stroke depression category before (1a) and after (1b) taking into account the baseline level of tobacco dependence. Model 2. Impact three months follow-up variables on post-stroke depression category before (2a) and after (2b) taking into account the level of tobacco dependence.

*FTND* Fagerström Test for Nicotine Dependence, *MoCA* Montreal Cognitive Impairment, *NIHSS* National Institutes of Health Stroke Scale, *mRS_hosp* baseline modified Rankin Scale collected at the end of hospitalization.

In Model 1, adding the level of tobacco dependence (model 1b) to the known PSD risk factors (age, gender, stroke severity at admission, history of depression; model 1a) improved significatively the model (*X*^2^ = 5.35; *p* = 0.021). Baseline levels of tobacco dependence and female gender were independently associated with a greater risk of PSD (respectively *OR* 1.58, 95% *CI* 1.03–2.41; *OR* 14.59; 95% *CI* 1.46–146.08), but not the other risk factors.

In Model 2, adding the level of tobacco dependence (model 2b) to the known PSD risk factors (age, gender, level of functional independence at follow-up, cognitive functioning at follow-up, history of depression; model 2a) improved significatively the model (*X*^2^  = 7.57; *p* = 0.006). Baseline levels of tobacco dependence and female gender were independently associated with a higher risk of PSD (respectively *OR* 1.97, 95% *CI* 1.11–3.52; *OR* 19.58, 95% *CI* 1.26–303.48). This model was not significantly improved when the “evolution of substance” variable was added (*X*^2^  = 1.83; *p* = 0.171).

## Discussion/conclusion

Tobacco, alcohol and cannabis consumption are well-known stroke risk factors and potential causes of addiction disorders but, to date, the influence of their severity on post-stroke emotional outcome has been neglected. The main results of this prospective study are the following: (i) about 30% of the stroke population had a clinically defined problematic substance use, with a predominance of problematic tobacco use (ii) half of the patients with a problematic substance use at admission presented a PSD at three months post-stroke (iii) the severity of tobacco dependence was revealed as an independent risk factor of PSD (iv) evolution in substance use did not seem to increase the risk of PSD.

Stroke patients are routinely asked about their consummatory behaviors in clinical practice and the frequency of current smokers, alcohol drinkers and, more recently, cannabis users are regularly reported in the demographic characteristics of stroke cohorts. While the association between the intensity of substance use (quantities) and the risk of stroke^19,20^ has been demonstrated, the level of dependence to these substances is almost never reported though it might be a major limit in the quitting process that is systematically recommended in stroke secondary prevention. Using standardized clinical evaluations, the present study highlights the high prevalence of problematic substance use in this first-ever stroke population, with notably over 20% of our cohort presenting a tobacco dependence. This result emphasizes the need to conduct a deeper evaluation of these addictive behaviors in daily practice in order to set up specific management and provide better support to allow patients to strike out these risk factors.

In addition, this study suggested for the first-time that stroke patients with a clinically defined problematic substance use might constitute a sub-group with a higher vulnerability to present post-stroke mood impairments. Strikingly, more than half of our sample was categorized with a PSD at follow-up, a rate that is also much higher than the 30% reported in larger stroke cohort studies that used similar CESD cut-offs^21^. Moreover, about 30% of our sample had a severe PSD, suggesting that these patients are prone to develop severe symptoms that may in turn contribute to more pharmaco-resistant depression and a poorer functional outcome in the long term.

In the population without previous history of stroke, substance use disorder, including nicotine dependence, has been previously demonstrated as a risk factor for depression after controlling for sociodemographic characteristic and psychiatric comorbidity^9,22,23^. Importantly, the co-occurrence of depression and substance abuse is known to be high, a condition that has been defined as a dual diagnosis, where both disorders have bi-directional relationships, mutually reinforcing each other^24^. According to the recent review commissioned by the NICE Centre for Public Health in UK, individuals with a dual diagnosis experience some of the worst health, have increased levels of unmet treatment needs and are among the most vulnerable in society^25^. These comorbidities could relate to the so-called self-medication hypothesis, where substances are used by the patients to cope with their psychological distress^26,27^. Facing the physical and psychological impact of stroke, these patients are thus exposed to a ‘double’ treatment challenge when asked to follow the secondary prevention recommendation. Therefore, an early identification of problematic substance use might help clinicians to provide appropriate intervention and follow-up to prevent post-stroke mood disorders and favor stroke secondary prevention. Evaluation of the severity of depressive symptomatology during the acute phase should also be part of the standard evaluation for these patients as, independently of stroke, those with more severe symptoms have a higher risk to fail their consumption cessation attempt^28^.

When we focused on the sub-sample of patients with a problematic tobacco use, we found an independent association between the severity of tobacco dependence at baseline and the risk of presenting a PSD at three months. Among PSD risk factors, female gender, stroke severity and aphasia are the most consistent across publications while, to the best of our knowledge dependence to substance use has never been investigated. According to the review by Kutlubaev and Hackett^7^, among 23 studies on PSD, only two evaluated the association of alcohol and tobacco consumption with PSD. Neither identified an independent role for these factors, which might indicate the specific role of dependence over simple consumption. In a more recent meta-analysis of PSD risk factors, tobacco consumption significantly predicted PSD (mean *RR* 1.36, 95% *CI* 0.86–2.16) but not alcohol consumption (mean *RR* 0.83, 95% *CI* 0.49–1.42)^11^. Of note, if we confirmed the close relationship between gender and PSD, we failed to identify any independent association with stroke clinical severity (as indexed at baseline by the NIHSS, and at three months of follow-up by the mRS). The selection of subjects able to fill the different questionnaires excluded more severe patients and those with aphasia, which could explain this result.

The mechanism underlying the association between problematic tobacco use and PSD is still a matter of debate^29^. Here, the fact that the severity of tobacco dependence doubled the odds of PSD beyond the impact of prior psychological status suggests that additional mechanisms are involved in this association. Dependence to tobacco might also contribute to a brain frailty through structural or molecular brain changes, including dopaminergic or serotoninergic pathways that might increase the risk of PSD^30^.

Surprisingly, while we expected that the favorable evolution in substance use to achieve a better life-style in patients unprepared to quit might contribute to increase their level of acute anxiety and subsequent depressive symptoms^31^, we failed to observe such an effect on the association between the level of tobacco dependence and PSD. Besides being potentially explained by power limitations, this expected deleterious effect might also have been counter-balanced by the strong motivation to quit related to the fear of stroke recurrence. Some studies even reported a reduced severity of depressive symptoms among patients who successfully stopped tobacco consumption^32^, suggesting of positive reinforcement effect.

The results of the present exploratory study should be interpreted cautiously due to several limitations. First the sample size is relatively small and the predictive results apply to patients with a problematic tobacco use while for those with problematic alcohol use or problematic cannabis use more prospective data will have to be collected. Second, our sample does not reflect the overall stroke population that have been included in hospital-based studies due to the exclusion of more severe and aphasic patients, leading to a lack of variance of stroke severity scores. However, patients with low severity at baseline are often those for whom invisible handicap, such as a mood disorder, is neglected and underdiagnosed; therefore, the identification of specific risk factors such as problematic substance use might represent a true improvement in their management. Third we used a self-report to defined PSD and similar CESD cut-offs for men and women while recent studies suggested that gender related cut-off might be more appropriate. However, gender was accounted for in our analyses.

In conclusion, this exploratory study reports for the first-time in a non-severe first-ever stroke hospital-based population the high frequency and potential deleterious consequences of addictive behaviors on post-stroke mood outcome. To draw firm conclusions on this issue, and to optimize post-stroke management in the long run, additional studies with larger sample size using standardized clinical screening tools will be needed. Moreover, the validity of the screening tools we used will need to be assessed among more severe stroke patients.
